# Optogenetic restoration of retinal ganglion cell activity in the living primate

**DOI:** 10.1038/s41467-020-15317-6

**Published:** 2020-04-03

**Authors:** Juliette E. McGregor, Tyler Godat, Kamal R. Dhakal, Keith Parkins, Jennifer M. Strazzeri, Brittany A. Bateman, William S. Fischer, David R. Williams, William H. Merigan

**Affiliations:** 1 Center for Visual Science, University of Rochester, 601 Elmwood Ave, Box 319, Rochester, NY 14642 USA; 2 Institute of Optics, University of Rochester, Wilmot Building, 275 Hutchison Road, Box 270186, Rochester, NY 14627-0186 USA; 3 David & Ilene Flaum Eye Institute, University of Rochester Medical Center, 601 Elmwood Avenue, Box 659, Rochester, NY 14642 USA

**Keywords:** Retina, Preclinical research, Imaging and sensing

## Abstract

Optogenetic therapies for vision restoration aim to confer intrinsic light sensitivity to retinal ganglion cells when photoreceptors have degenerated and light sensitivity has been irreversibly lost. We combine adaptive optics ophthalmoscopy with calcium imaging to optically record optogenetically restored retinal ganglion cell activity in the fovea of the living primate. Recording from the intact eye of a living animal, we compare the patterns of activity evoked by the optogenetic actuator ChrimsonR with natural photoreceptor mediated stimulation in the same retinal ganglion cells. Optogenetic responses are recorded more than one year following administration of the therapy and two weeks after acute loss of photoreceptor input in the living animal. This in vivo imaging approach could be paired with any therapy to minimize the number of primates required to evaluate restored activity on the retinal level, while maximizing translational benefit by using an appropriate pre-clinical model of the human visual system.

## Abstract (web summary)

Non-human primate models are important for the development of high quality vision restoration therapies for blindness. Here, the authors demonstrate restoration of light responses in foveal retinal ganglion cells of the living macaque following optogenetic gene therapy.

## Introduction

Optogenetic therapies aim to restore light sensitivity in postreceptoral retinal cells of patients with irreversible sight loss caused by photoreceptor degeneration. In vivo preclinical testing is typically performed in mice^1–3^, and while these animals provide access to genetic models of retinal disease, they have different immune function and retinal physiology to humans. Only the primate has a human-like fovea, the retinal structure which mediates high acuity central vision and dominates our visual experience. Studies of vision restoration in primate tissue have previously been limited to electrophysiological recording of retinal ganglion cell (RGC) responses in excised tissue^4,5^.

Using adaptive optics scanning light ophthalmoscopy (AOSLO) calcium imaging we present in vivo evidence that optogenetic therapy restores RGC responses in the fovea of the living primate. We are able to drive the same foveal RGCs cells either optogenetically or through their normal photoreceptor pathway and compare the activation patterns produced. Finally we demonstrate that optogenetic activation of RGCs remains possible two weeks after photoreceptor ablation in the living primate. This in vivo optical stimulation and imaging platform can be used to evaluate any vision restoration strategy at a pre-clinical stage, informing and refining which therapies enter human clinical trials.

## Results

### Recording optogenetic activation of foveal RGCs in vivo

**Fig. 1:**
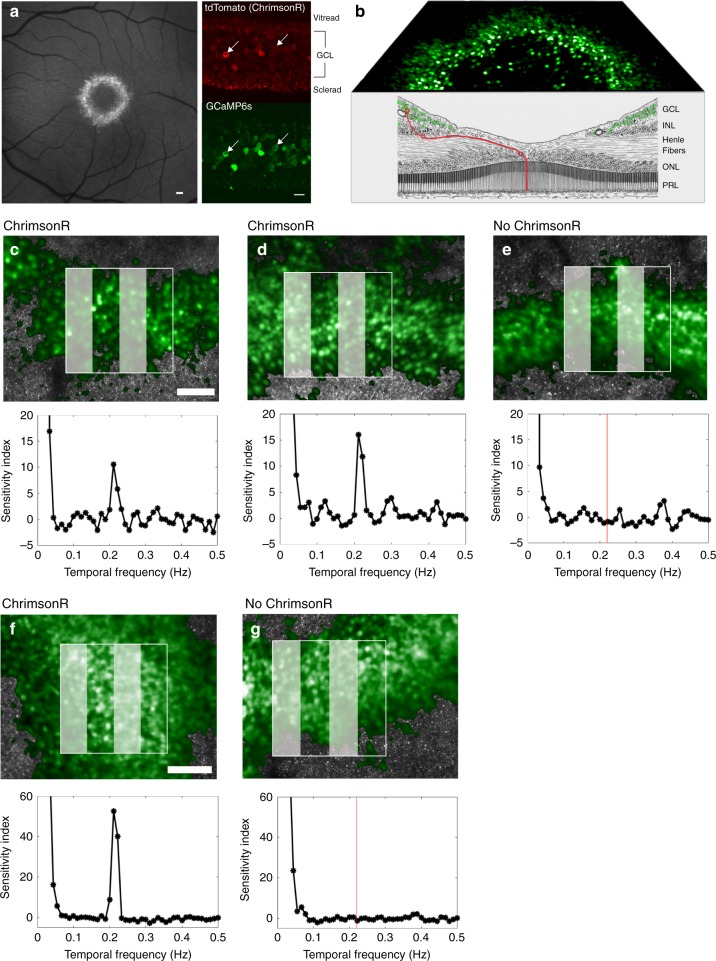
GCaMP6s recording from foveal retinal ganglion cells in the living macaque shows ChrimsonR mediated responses to a drifting grating stimulus. **a** (Left panel) Scanning light ophthalmoscope image of GCaMP6s expression in the ring of ganglion cells serving the foveal cones, scale bar 150μm. (Right panel) Confocal microscope images of GCaMP6s and ChrimsonR co-expression, scale bar 20μm. **b** Schematic diagram of the fovea, (modified from Polyak, 1941)^24^ showing fluorescent foveal retinal ganglion cells (green) laterally displaced from their photoreceptor receptive fields (red line). **c** (Upper panel) Adaptive optics scanning light ophthalmoscope image of the stimulated region of retina from which the data shown in the lower panel is derived. Scale bar 100 μm. Fluorescent retinal ganglion cells shown in green, photoreceptor mosaic in gray. (Lower panel) Mean response of 48 cells to 0.2 Hz patterned stimulus in the left eye of animal 2, 5 months after intravitreal injection of ChrimsonR and GCaMP6s. **d** Mean response of 38 cells to 0.2 Hz patterned stimulus in the right eye of animal 2, 10 months after intravitreal injection of ChrimsonR and GCaMP6s. **e** There is no response in the left eye (38 cells) of the control GCaMP6s-only animal (animal 3) at 0.2 Hz (red line) 5 months after intravitreal injection of GCaMP6s. **f** Mean response of 65 cells to 0.2 Hz patterned stimulus in the left eye of animal 4, 7 weeks after intravitreal injection. **g** No response in the right eye of animal 4 (49 cells) at 0.2 Hz (red line) that has received GCaMP6s only, 9 weeks after intravitreal injection. Representative results are shown. Data was taken with similar results in more than 20 separate imaging sessions for the treated animals and across 6 imaging sessions for the control animals. Source data are provided as a Source Data file.

This study required both optical stimulation of, and optical recording from, the cells of the inner retina in the living macaque. Intravitreal co-injection of two adeno-associated viruses (AAV2), both containing a ubiquitous CAG promoter, produced co-expression of both the optogenetic actuator ChrimsonR^6^, and the calcium indicator GCaMP6s^7^ in the same RGCs (Fig. 1a). Consistent with previous studies, expression was confined to a ring of RGCs on the margins of the foveal pit, where the inner limiting membrane is thinnest^8,9^. In the fovea RGCs are laterally displaced from the photoreceptors that drive them (Fig. 1b), making it possible to activate RGCs either by direct optogenetic stimulation, or through their normal cone inputs, using spatially localized stimuli applied either to the ganglion cells themselves or to their photoreceptors. Figure 1c, d, f shows ChrimsonR mediated responses to a spatially localized 0.2 Hz drifting grating focused onto the RGC layer in three eyes, 5 months, 10 months and 7 weeks after intravitreal injection of the ChrimsonR therapy.

The absence of any RGC response when the grating stimulus is applied directly to ganglion cells in two control eyes expressing GCaMP6s only, and not ChrimsonR (Fig. 1e, g) demonstrates that responses observed in the treated eyes were not the result of light scattered onto photoreceptors. The cells in the control eyes were otherwise normally responsive to photoreceptor stimulation (Supplementary Fig. 1). Histograms of the Fourier amplitude at 0.2 Hz scaled by the mean intensity of each cell (F/F_0_) (Supplementary Fig. 2) show differing distributions of activity between the trial and control cases. The range of F/F_0_ responsivities may reflect differing levels of ChrimsonR expression amongst individual cells.

### Optogenetic vs photoreceptor driven activity in the same RGCs

**Fig. 2:**
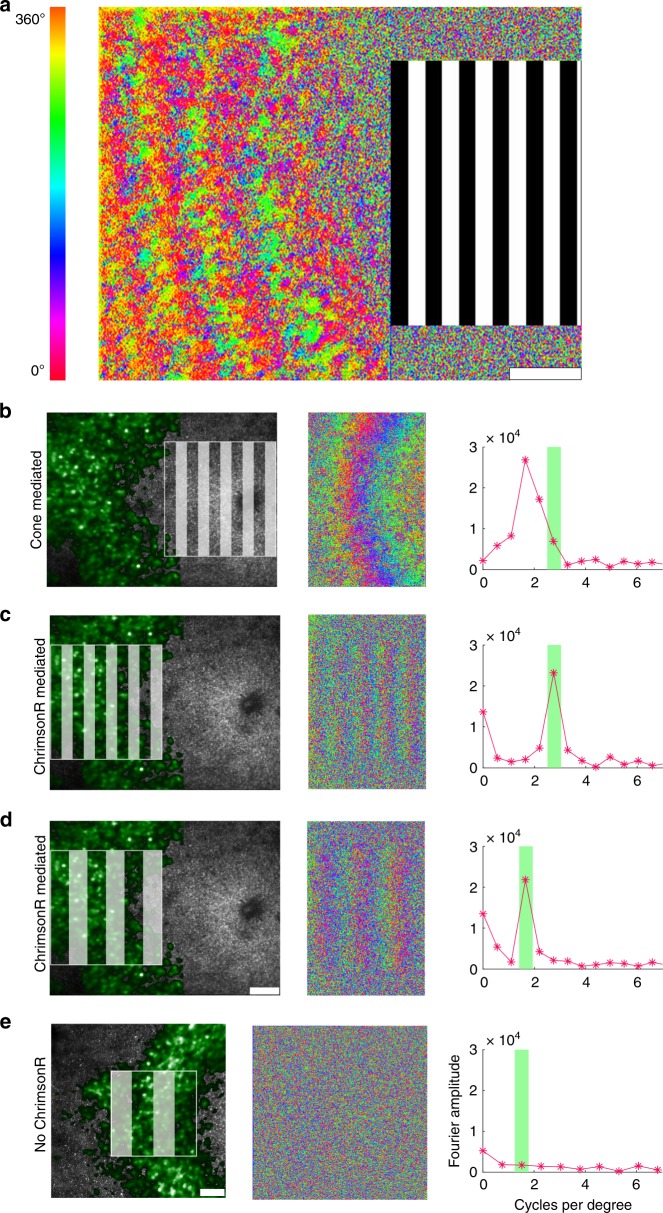
Optogenetic therapy restores characteristic retinal ganglion cell responses to patterned stimuli in the living primate. **a** Pixelwise map of the temporal phase of ganglion cells responding to a 0.2 Hz drifting grating presented to foveal cones. **b** The spatial frequency of the ganglion cell layer (GCL) response to a 2.7 cycles per degree stimulus presented at the fovea. The spatial frequency of the response is lower than the spatial frequency of the stimulus because of the anatomical expansion of the ganglion cell density relative to the density of the foveal cones to which they are connected. **c** The spatial frequency of the ChrimsonR mediated ganglion cell response to a 2.7 cycles per degree stimulus applied directly to the ganglion cell ring matches the spatial frequency of the stimulus. **d** The spatial frequency of the ChrimsonR mediated GCL response to a 1.7 cycles per degree stimulus exactly matches the spatial frequency of the stimulus. This lower spatial frequency stimulus applied to the GCL mimics the natural photoreceptor mediated response to the higher spatial frequency shown in (**b**). **e** Applying a 1.1 cycles per degree stimulus directly to the GCL in the control animal, which did not receive ChrimsonR treatment, elicits no spatial response. All scale bars 100 μm. Depending on the available light budget, trials were repeated up to three times in the same location within a single imaging session and produced similar results.

The RGC response to patterned stimuli produced by direct activation of ChrimsonR was also different to, and easily distinguishable from, excitation via the normal cone pathway. The spatial pattern of activation can be seen in the variation in the phase of response across the RGC array as shown in Fig. 2a. When a grating was presented to photoreceptors at the foveal center (Fig. 2b), the spatial frequency of the response was 2.5 times lower than the spatial frequency of the grating itself and the phase pattern was curved, consistent with the anatomy of the fovea^10^. By contrast, the spatial frequency and shape of the ChrimsonR mediated RGC response matched those of the applied stimulus precisely, (Fig. 2c, d). No response was observed in the control eye which did not contain ChrimsonR (Fig. 2e).

**Fig. 3:**
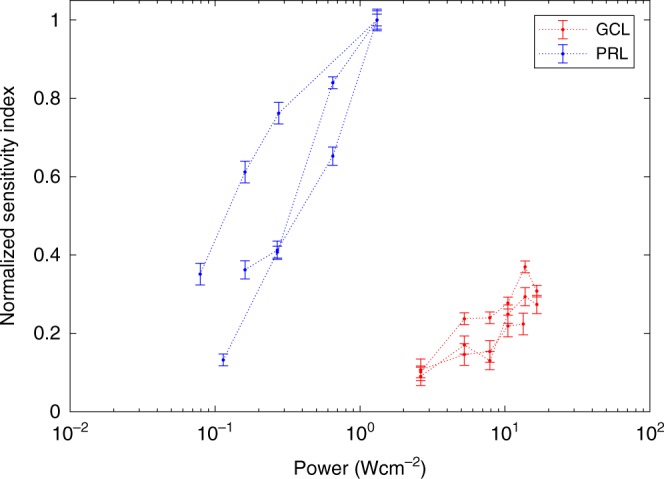
Comparison of photoreceptor mediated RGC activity (blue) and ChrimsonR mediated RGC activity (red) over a range of stimulus powers. Spatially localized grating stimuli were focussed on the photoreceptor layer (PRL), or directly on the ganglion cell layer (GCL). Mean cellular response was quantified as the sensitivity index normalized to the maximum photoreceptor response for that ascending staircase. Dashed lines connect data from the same ascending staircase. Two data sets were recorded from 128 cells, 51 weeks after injection, and the third from 90 cells 61 weeks after injection. To achieve the same level of RGC activity in this high light regime, the optogenetic stimulus had to be two orders of magnitude more intense than the photoreceptor stimulus. Data are presented as mean values +/− standard deviation. Source data are provided as a Source Data file.

To assess the relative sensitivity of ChrimsonR mediated, compared to photoreceptor mediated, stimulation, the same RGCs were stimulated via each pathway at a series of light intensities. Average cellular responses were computed from data taken 12 and 14 months after intravitreal injection and are shown in Fig. 3, normalized to the maximum photoreceptor driven response. These data were collected in the presence of the bright 488 nm imaging light that, while not directly incident on the cones tested, likely adapted them into the upper photopic range. In these conditions approximately a hundred-fold increase in power was needed to achieve a similar response amplitude using optogenetic stimulation compared to stimulation of the same cells through the photoreceptor pathway.

### Optogenetic responses persist after photoreceptor ablation

**Fig. 4:**
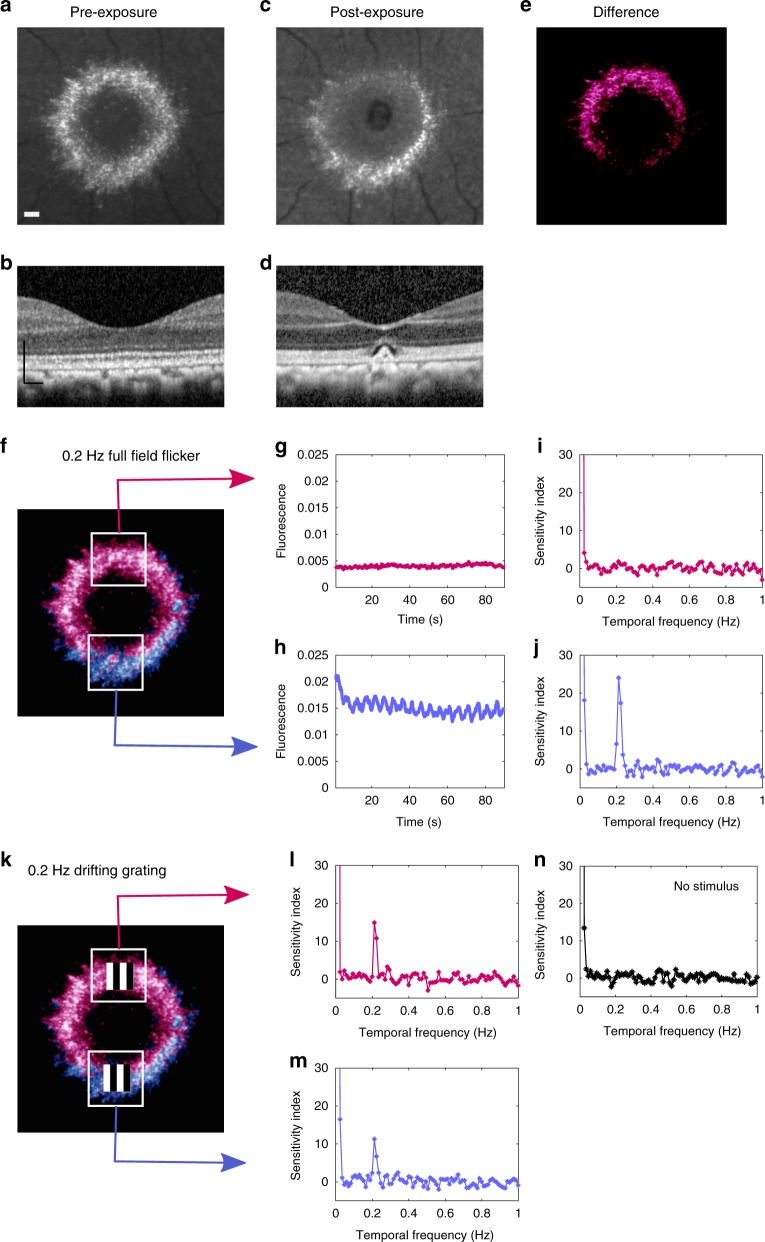
ChrimsonR mediated responses can be recorded from cells that have lost their photoreceptor input, restoring light sensitivity. **a** Confocal SLO image of GCaMP6s fluorescence in foveal RGCs pre-scotoma. 150 µm scale bar also applies to (**c**, **e**, **f**) and (**k**). **b** OCT image pre-scotoma 150 µm scale bar also applies to (**d**). **c** Confocal SLO image of GCaMP6s in foveal RGCs post-scotoma, lesion appears as dark region in the superior fovea. **d** OCT image post-scotoma showing damage to the photoreceptor layer. **e** Difference between images (**a**) and (**c**), highlighting the putative region of RGCs without photoreceptor input. **f** Pseudo-colored GCaMP6s image showing recording areas in the superior fovea with damaged photoreceptor input (pink) and inferior region with photoreceptor input intact (purple). **g** No periodic response to 0.2 Hz pan-retinal visual stimulus from 46 RGCs in superior region, indicating a loss of photoreceptor input. **h** Periodic response from 48 RGCs in the inferior imaging area, indicating normal photoreceptor input. **i** Temporal Fourier transform of data shown in (**g**) showing no response at 0.2 Hz consistent with loss of photoreceptor input**. j** Temporal Fourier transform of data in (**h**) showing a response at 0.2 Hz consistent with normal photoreceptor input. **k** As (**f**), with positions of spatially localized high power grating stimuli. **l** Fourier transform showing optogenetic RGC response to a 0.2 Hz spatially localized stimulus despite the loss of photoreceptor input. **m** Fourier transform showing optogenetic RGC response to a 0.2 Hz spatially localized stimulus. **n** Control, spatially localized constant mean luminance presented to the superior region, no periodic response at 0.2 Hz demonstrating the signal in (**l**) is dependent on the visual stimulus. This data was collected at a single time point using the maximum light budget available two weeks after photoreceptor ablation and therefore no repeat measurements could be performed in this case. Source data are provided as a Source Data file.

To test the ability of ChrimsonR to restore function when normal visual input is lost, photoreceptor input to the imaged cells was eliminated by exposing a patch of cones in the superior fovea to a high intensity pulsed femtosecond laser delivered through the adaptive optics system. Histology demonstrated that these exposure parameters cause complete loss of photoreceptors in macaque retina (Supplementary Fig. 3). Scanning light ophthalmoscopy (SLO) and optical coherence tomography (OCT) images pre and post exposure are shown in Fig. 4a–e; a reduction in GCaMP6s fluorescence emission was observed in the superior portion of the RGC ring consistent with the loss of photoreceptor mediated activity evoked by the imaging light. Pan-retinal flicker stimulation at an intensity sufficient to activate photoreceptors but insufficient to activate an optogenetic response, confirmed that the upper portion of the foveal RGC ring had lost photoreceptor input (Fig. 4f–j). ChrimsonR mediated responses however, were maintained in RGCs measured two weeks after the cells were deprived of photoreceptor input, at comparable levels to ChrimsonR mediated RGC responses from the unaffected inferior fovea (Fig. 4k–m).

## Discussion

The results of this study advance the possibility of successful optogenetic vision restoration in humans by showing that a virally inserted optogenetic actuator can restore light driven RGC responses to patterned stimuli in the fovea of the living primate. Optogenetics holds special promise as a vision restoration therapy as it does not require the complex surgery necessary to implant electrical prostheses or stem cells, but rather foveal cells can be made light sensitive using a single intravitreal injection. The safety record of AAV delivery in gene therapy is well established in humans^11^ and work is ongoing to achieve pan-retinal transduction by intravitreal injection^12^. By rendering post-receptoral cells individually light sensitive, this therapy overcomes the limited electrode density of current electrical prostheses and offers the promise of high acuity restored vision at the fovea. Adopting a restorative biological approach rather than electrical or chemical prostheses, optogenetic therapy has the potential to be more stable, less toxic and easier to deploy in humans.

Optogenetic therapies are in early clinical trials, however, pre-clinical development remains critical to ensure that the therapy is effective as well as safe. Testing efficacy in patients with a complicated natural history and years of vision loss represents the most realistic but the most challenging environment to evaluate and improve therapies. By performing pre-clinical studies in primate in vivo we have simplified the situation, acutely removing photoreceptor input to RGCs and recording optogenetic function two weeks after photoreceptor function is lost. In vitro studies of optogenetic therapy in primate involved pharmacological blockade of photoreceptor input immediately before recording, so it has remained unclear to what extent medium term changes in the status of RGCs following loss of photoreceptor input reduce the effectiveness of all vision restoration therapies. Success at two weeks is promising and longer term investigation of the stability and efficacy of the treatment should now be achievable with a relatively small number of animals. By studying restoration at the retinal level in vivo we can directly observe the intact circuits that are being acted on by the therapy rather than the end result, making problems easier to diagnose and overcome.

To compare relative responsivity of foveal RGCs driven by ChrimsonR versus normal photoreceptor stimulation, we used drifting gratings to drive the same group of cells via each of the two pathways, demonstrating important differences between optogenetic and photoreceptor mediated activity at the retinal level. Figure 2 demonstrates that adjustment of the spatial frequency of the stimulus will be required to mimic the RGC activation pattern evoked by natural photoreceptor stimulation in the fovea. Figure 3 shows that the ChrimsonR pathway required two orders of magnitude higher power to achieve a similar level of activation as photoreceptor stimulation. It should be noted that our experiments were conducted in a high light photopic regime and whilst the 488 nm light needed for calcium imaging is not directly incident on the photoreceptors, scattered light is likely to adapt them. As such this comparison represents a snapshot of the relative activity driven by the optogenetic channels versus photoreceptors in these experimental conditions and is not a full characterization of their relative sensitivities as a function of adaptation level. Cone photoreceptors are notable because of their large dynamic range and therefore in low light conditions the difference between photoreceptor mediated and optogenetic sensitivity would be much larger than observed here. We stress that the absolute intensity thresholds for activation were not compared in this study, but the literature suggests these values differ even more substantially. More sensitive mammalian opsins^13^ and light sensitive glutamate receptors^2,3^ are becoming available as alternatives to optogenetic actuators based on relatively insensitive microbial opsins, however, these actuators typically have lower saturation points, and cannot yet cover the greater than 5 log unit dynamic range of primate cones^14^. In future experiments lower imaging light levels may be possible, allowing us to probe relative sensitivities without strong cone adaptation.

We have demonstrated efficacy of optogenetic therapy in the living primate at the retinal level, an early stage in the visual pathway, however, it will be important to establish how these restored signals are interpreted by downstream nuclei of the visual system, as the level of visual plasticity in the adult primate is finite^15^. There are important differences between ‘normal’ ganglion cell responses that are generated by photoreceptors verses those evoked by direct optogenetic activation of ganglion cells themselves. By making RGCs intrinsically light sensitive we are bypassing the normal adaptation and gain control mechanisms conferred by bipolar, horizontal and amacrine cells. One impact of this is the loss of ‘off centre’ ganglion cells which respond to light decrements and are believed to increase dynamic range and contrast sensitivity^16^. Optogenetically restored responses would all become ‘on centre’ unless actuators could be inserted into bipolar cells or remaining photoreceptor inner segments. The impact of these physiological changes on visual performance is poorly understood and must ultimately be tested psychophysically.

The animals used in this study received immune suppression prior to and following intravitreal injection. In animal 2 this was stopped after 9 months with no acute loss of ChrimsonR. The impact of the immune response on the efficacy and stability of therapeutic interventions in primates is still little understood. Given the potentially serious side effects associated with immune suppressants it is desirable to minimize their use and further studies are necessary to establish whether a brief period of immune suppression during the period of viral infection is adequate or even necessary to achieve strong and stable expression. An in vivo imaging approach makes these pre-clinical longitudinal studies more readily achievable and is of particular importance in primate studies where the immune system is highly variable between individuals.

We demonstrated optogenetic responses from inner retinal neurons 14 months after intravitreal injection of the ChrimsonR construct and two weeks after acute loss of photoreceptor function. The ability to perform in vivo longitudinal monitoring opens up the possibility of testing how the loss of sensory input affects primate RGC responsivity in the long term. This is critical because spontaneous hyperactivity^17,18^ and structural remodeling^19^ have been reported in the inner retina following photoreceptor loss. Understanding how these changes alter restored function and how to mitigate them^20^ is crucially important to all vision restoration therapies. The combination of optogenetic stimulation, functional readout in the intact eye, and the ability to cause localized acute damage now makes these studies possible in primates.

A limitation of the calcium imaging approach applied here is the relatively long time constant of GCaMP6s at 0.6s^7^. This makes assessing the temporal limits of optogenetically restored visual responses challenging. In this study visual stimuli are delivered through the 25 Hz AOSLO system which is then modulated by a 0.2 Hz envelope in the form of a drifting grating. This allows us to use Fourier methods to quantify the GCaMP6s signal evoked by the periodic visual stimulus. Increasing the temporal frequency of the envelope would decrease the measured response because the calcium response would not return to baseline before stimulation occurred again. This means that changing the frequency of the envelope gives insight into the calcium dynamics rather than the temporal limits of optogenetic vision restoration. Whilst the development of voltage indicators may allow high speed functional imaging in future studies, at present temporal limits of optogenetically restored activity are better explored using electrophysiology or psychophysics.

As the fovea is the seat of high acuity vision, the techniques introduced here could be used to explore the upper limit of spatial acuity that can be supported by optogenetics at the retinal level. We anticipate that acuity will be reduced relative to normal human vision as the light detectors are no longer individual cones but rather RGC somas including their dendritic trees, which are larger than the optical point spread function of the eye. This aspect of visual acuity could be evaluated using calcium imaging AOSLO. Additional factors may further reduce acuity including the loss of the off-center response, the jitter in the locations of ganglion cells in their array compared with the relative locations of the cones that drive them, reduced light sensitivity, the ability of the eye to focus the retinal image on the ganglion cell layer, and the fact that, unlike the cone photoreceptor mosaic, ganglion cells are distributed in three spatial dimensions instead of two. Visual psychophysics will therefore be the ultimate arbiter of the achievable acuity limit at the perceptual level.

It is important to note that vision resulting from optogenetic restoration of RGC activity at the fovea will be complicated by the displacement of the foveal RGCs into a ring around the central foveal photoreceptors. Visual stimuli falling on the ring will, at least initially, be interpreted as having originated from the foveal photoreceptor mosaic, and this will generate warping and perceptual distortion which may affect the usability of the restored vision. The data shown in Fig. 2 suggests that a visual stimulus presented directly to the RGC ring will be perceived as being 2.5 times smaller than it really is, because RGCs cover an expanded area relative to their original receptive fields in the cone mosaic. Additionally, there will be a 1° (radius) blind spot over the foveola where RGCs are absent. It may be possible to overcome these issues if stimuli can be pre-warped and presented to the eye using a head set with high resolution eye tracking. Head mounted eye trackers currently have poor performance which would make stabilization of the image potentially a key determinant of spatial acuity. It is also possible that patients will learn to adapt to the warping such that it no longer limits their visual performance. Visual psychophysics with awake behaving primates may allow us to understand how these distortions in shape and scale affect visual performance.

## Methods

### Animal care

The primates were socially housed in an AAALAC accredited institution. The monkeys had free access to water and food, providing a complete nutritious diet. In addition to daily food and water, monkeys were given various treats such as nuts, raisins and a large variety of fresh fruit and vegetables. An animal behaviorist provided a novel enrichment item to each monkey once a week which included items such as grapevines, fresh wheat grass and treat filled bags. Daily primate enrichment included 2–4 pieces of manipulata, a mirror, puzzle feeders rotated among all animals, daily movies or music and rotating access to a large, free ranging space with swings and elevated perches. They were cared for by the Department of Comparative Medicine which included four full-time veterinarians, five veterinary technicians, and animal care staff who monitored the health of the primates and checked for signs of discomfort at least twice daily. This study was carried out in strict accordance with the Association for Research in Vision and Ophthalmoscopy (ARVO) Statement for the Use of Animals and the recommendations in the Guide for the Care and Use of Laboratory Animals of the National Institutes of Health. The protocol was approved by the University Committee on Animal Resources of the University of Rochester (PHS assurance number: D16-00188(A3292-01)).

### Immune suppression

Immune suppression with Cyclosporine A was begun one week prior to intravitreal injection at a starting dose of 6 mg kg^−1^ delivered sub-cutaneously. Blood trough levels were collected weekly to titrate the dose into a therapeutic range of 150–200 ng ml^−1^ and then maintained at that level. Animal 2’s body condition score began to drop after 9 months so immune suppression was stopped in that case.

### Co-expression of ChrimsonR and GCaMP6s

*AAV2-CAG-tdTomato-ChrimsonR* and *AAV2-CAG-GCaMP6s*, synthesized by the University of Pennsylvania vector core were intravitreally injected into four eyes of three normal *Macaca fascicularis* as described previously^8^. Briefly, the eye was sterilized with 50% diluted betadine before the vector was injected into the middle of the vitreous at a location approximately 3 mm behind the limbus using a tuberculin syringe and 30 gauge needle. Two additional control eyes received an intravitreal injection of *AAV2-CAG-GCaMP6s* only and no ChrimsonR. The neutralizing antibodies, injected titres, volumes and animal number corresponding to each eye are detailed in Supplementary Table 1. Neutralizing antibodies to AAV2 were 1:25 or lower in all four injected animals. Following injection each eye was imaged weekly with a conventional scanning light ophthalmoscope (Heidelberg Spectralis) using the 488 nm autofluorescence modality, to determine the onset of expression, image quality and to monitor eye health. Animal 2 and the control animal received 50 µl of triamcinolone (Kenalog-40) 3 weeks following the injection to treat the symptoms of uveitis. A fundus camera (Topcon TRC 50ex) equipped with custom filters to spectrally separate GCaMP6s (excitation 466/40 nm, emission 520/28 and tdTomato (excitation 549/25 nm and emission 586/20 nm) were used to monitor expression levels independently.

### Histology

Animal 1 was euthanized with intravenous pentobarbital to effect, perfused with 1 litre heparinized saline and 2 litres of 4% paraformaldehyde. 100 µl of additional fixative was injected directly into the vitreous humor. The eye was enucleated, and the retina removed from the eyecup and postfixed in 4% paraformaldehyde for 2 h before being placed in 10%, followed by 30%, sucrose cryoprotectant until equilibrated. The tissue was flash frozen and an ultramicrotome used to cut the retina into 14 μm sections. Dried sections were coverslipped with vectorshield containing DAPI and examined under the confocal microscope, to image GCaMP6s expression (excitation 488 nm, emission 530/43 nm) and tdTomato (543 nm excitation, 620/52 nm emission), denoting expression of ChrimsonR.

To assess the extent of photoreceptor loss caused by the ultrafast laser exposure delivered to the retina through the adaptive optics system, animal 5 was euthanised 4 weeks following the exposure as described previously and perfused with 2.5% glutaraldehyde and 4% paraformaldehyde. The eye was enucleated and the tissue postfixed and dehydrated before plastic embedding and sectioning into 2.5 μm sections. Full details of the protocol can be found in Walters et al.^23^. A two part hematoxylin and eosin stain was performed to label nuclei blue and cytoplasm pink, allowing assessment of structural damage.

### Animal preparation for imaging

All monkeys were fasted from 4–18 h prior to anaesthesia induction. Anaesthesia induction began with 10 mg kg^−1^ Ketamine, 0.25 mg kg^−1^ Midazolam, and 0.017 mg kg^−1^ Glycopyrrolate intramuscularly. The monkey was then given 5 mg kg^−1^ Ketofen intra-muscularly to prevent pain or inflammation from the lid speculum being placed in the eye during imaging for an extended period. The pupil was dilated with a combination of Tropicamide 1% and Phenylephrine 2.5%. In cases of minimal pupil dilation within the standard time, Phenylephrine 10% and/or Cyclopentolate 1% drops were administered. Both eyes were covered with a hydrating ophthalmic gel (Genteal). The target eye then had the lid speculum placed to keep the eye open during imaging and a contact lens was placed to ensure corneal protection. The fellow eye was taped closed with porous tape, to protect the cornea from drying.

The animal was placed in a stereotaxic cart. Prior to intubation, an oxygen mask with 1–2% isoflurane, was placed over the monkey’s face to allow for adequate sedation for intubation. An intravenous drip of Lactated Ringers with 5% Dextrose was maintained at 5 ml kg^−1^ h^−1^ for the duration of imaging. The monkey was intubated and maintained at a surgical plane of anaesthesia with Isoflurane 1.0–2.5%. A Bair Hugger warming system was placed over the monkey to maintain body temperature. Monitoring devices including, rectal temperature probe, blood pressure cuff, electrocardiogram leads, capnograph, and a pulse oximeter, were used to ensure proper monitoring of all vitals. Temperature, heart rate and rhythm, respirations and end tidal CO_2_, blood pressure, SPO_2_ and reflexes were monitored consistently and recorded every fifteen minutes.

After a surgical plane of anaesthesia had been established, the monkey was given a 300 mcg kg^−1^ bolus of Rocuronium that was mixed to a concentration of 800 mcg ml^−1^, followed by an intravenous infusion of 300 mcg kg^−1^ h^−1^. Once respirations ceased, the monkey was maintained on a ventilator until imaging was over and the infusion was turned off. Once a peripheral nerve response was established, an intravenous dose of Glycopyrrolate 0.01 mg kg^−1^ was given. Five minutes after the Glycopyrrolate, Neostigmine 0.05 mg kg^−1^ was given intravenously. The monkey was monitored for indications of breathing against the ventilator and then removed from the ventilator once able to breath without assistance. The monkey was removed from the Isoflurane no sooner than fifteen minutes after the Neostigmine injection to ensure stability off the ventilator. The monkey was then allowed to wake up and extubated once all reflexes had returned.

### Photoreceptor ablation by ultrafast laser exposure

To create a small scotoma suitable for testing restored vision in RGCs lacking photoreceptor input, a 0.87 × 0.79 degree patch of retina was exposed for 106 ms to a scanning, 55 fs pulsed 730 nm laser, with an average power of 4.48 W cm^−2^ and a repetition rate of 80 MHz. The exposure was delivered to the photoreceptor layer using an adaptive optics scanning light ophthalmoscope^21^. The structural impact of the exposure was assessed with OCT. SLO 488 nm imaging post-exposure was used to identify a region of reduced fluorescence providing a preliminary indication of ganglion cells that had been functionally impacted by photoreceptor damage. High resolution functional testing to assess the impact of the lesion was then conducted using the AOSLO as described in the following sections.

### AOSLO calcium imaging

Data was collected using an AOSLO system described in Gray et al.^22^. Briefly, a Shack-Hartman wavefront sensor and deformable mirror were used to correct aberrations in closed loop using an 843 nm laser diode source (Thorlabs). During each trial the AO correction was static to prevent any periodic signal changes, between trials the loop was closed to refresh the shape of the mirror. A 796 nm superluminescent diode light source (Superlum) was focused on the photoreceptor layer and reflectance images were collected using a 2 Airy disk pinhole at a rate of 25.6 Hz. Simultaneously a 488 nm laser source (Qioptiq) was focused on the ganglion cell layer to excite GCaMP6s fluorescence, which was detected in a 517/20 nm emission band. An 8 airy disc pinhole was used to maximize signal collection. The excitation light was presented only during the forward scan phase and filled the whole field except for experiments comparing the activation of ganglion cells through photoreceptor versus ChrimsonR activity, where the 488 nm imaging light was confined to the region of ganglion cell bodies and foveal photoreceptors were not exposed. The imaging light intensities used were 3.8 mW cm^−2^ in Fig. 1(c, d), 3 and Supplementary Fig. 1a, 4.3 mW cm^−2^ in Figs. 1e, 2e and Supplementary Fig. 1b, 4.5 mW cm^−2^ in Fig. 1f, g and 2.6 mW cm^−2^ in Figs. 2a–d and 4.

### Visual stimulation

To drive photoreceptors we presented a pan-retinal, temporally modulated LED stimulus in Maxwellian view (peak wavelength 590 nm, 0.2 Hz, mean luminance 0.75 mW cm^−2^). The stimulus was presented for 90 s following a 30 s period of adaptation to the imaging light. To drive ChrimsonR, a spatially localized, 561 nm 0.2 Hz square wave drifting grating stimulus was focused onto the ganglion cell layer using a laser presented through our 25.6 Hz scanning system. The drifting grating stimulus was generated by modulation of the intensity of this laser source creating grating pattern moving at 0.2 Hz. The stimulus was presented for 90 s following a 30 s period of adaptation to the imaging light and stimulus mean luminance. To compare photoreceptor and ganglion cell sensitivity the drifting grating stimulus was focused either at the photoreceptor layer and presented at the fovea or to the ganglion cell layer (GCL). The mean luminance of the visual stimulus presented during each trial was increased in a stepwise manner (an ascending staircase) to produce the data presented in Fig. 4. A spatial frequency of 1.1 cycles per degree and mean luminance of 12.5mWcm^−2^ was used in the trials presented in Figs. 1c, d, and 3, 14 mW cm^−2^ in Fig. 1e and 15 mW cm^−2^ in 1f–g. The mean stimulus luminances used in Fig. 2b–e were 0.9, 9.7, 9, and 10.8 mW cm^−2^ respectively. In each case the imaging light was focused at and localized to the foveal ganglion cell layer as described above. Control trials consisted of the presentation of a constant equivalent mean luminance for the duration of the trial or when light exposure was a concern in the case of the sensitivity comparison, the imaging light only. The stimulus was also presented in the same field of view without the imaging light to detect any optical bleed through, anti-stokes or tdTomato emission and this was subtracted from the test data in all cases except Fig. 3 where light exposure consideration limited the number of permissible trials. Additional data was also collected using a 640 nm stimulus to drive ChrimsonR confirming that responses were present in the absence of any tdTomato excitation (Data available on request). The visual stimuli and imaging fields were stabilized on the retina using an approach described previously^10^.

### Data analysis

To remove the effect of eye movements, each frame of the fluorescence video was co-registered using the corresponding high signal-to-noise infrared reflectance video. For each field of view, a single frame was chosen, typically the tenth infrared reflectance frame in the video, and frame to frame image registration of all videos for that field of view was performed using a whole frame cross correlation method. Frames were summed to create a fluorescence image of the ganglion cell layer and individual cells were segmented by hand to create a mask that could be applied to all videos with that field of view. All identifiable cells in the focal plane within the stimulation area were segmented. To illustrate the experimental paradigm in Figs. 1, 2, fluorescence images were contrast adjusted, thresholded, pseudo-colored and superimposed on the corresponding reflectance images. A similar process was used with SLO to illustrate the method in Fig. 4e, f, k. No such manipulations were performed on the raw data.

The frames corresponding to the adaptation period were removed from the registered fluorescence video and the segmentation mask was applied to the remaining frames. The mean of the signal within each cell mask was computed for each frame and a Hann windowing function was applied to the data. Each data sequence was temporally Fourier transformed into the frequency domain. The Fourier amplitudes were normalized relative to the standard deviation of the noise in the signal from 0.35 Hz to 0.55 and 0.65 to 1.1 Hz (avoiding the respiration rate) producing a response metric equivalent to the sensitivity index D’. This allowed comparison of data between different animals and different areas of the foveal ring. To produce the sensitivity comparison in Fig. 3, the same cell mask was applied to both the photoreceptor driven and ChrimsonR driven data in each ascending staircase. The sensitivity index characterising the magnitude of the response was computed as described and then both the optogenetic mediated and photoreceptor mediated data for each field of view was scaled by the magnitude of the maximum photoreceptor response. This allowed us to combine data sets from different areas of the foveal RGC ring and from different imaging sessions under the assumption that the photoreceptor response is constant. Three datasets from the right eye of animal 2 were combined to produce Fig. 4; two from the same imaging session from nasal and temporal sides of the ganglion cell layer 51 weeks after injection, and one from the nasal side at 61 weeks.

To assess the spatial frequency of the response, we Fourier transformed the raw fluorescence time course data on a pixel by pixel basis and from the result computed the phase of the response at 0.2 Hz for each pixel. The phase was assigned a color (rainbow color scheme ranging from 0 to 360 degrees as shown in Fig. 2) and phase maps of the response were produced (Fig. 2). To examine the spatial frequency of the response pattern more quantitively, the complex output from the pixelwise temporal Fourier transform, containing both the phase and amplitude signatures of the response, was spatially Fourier transformed. A two-dimensional Fourier transform was applied to data from the 255 × 255 pixel region of ganglion cells that were stimulated. The same region of interest was used in the photoreceptor stimulation condition. We observed low amplitude anti-stokes emission from the GCaMP6s in control trials with the stimulation laser only. While the amplitude of this signal was very low, the phase information contained in the signal was potentially misleading and therefore the Fourier transformed data for the stimulus only condition was subtracted prior to the production of the spatial Fourier transform.

### Reporting summary

Further information on experimental design is available in the Nature Research Reporting Summary linked to this paper.

## Supplementary information

Supplementary Information

Reporting Summary

## Data Availability

All raw data is available on request. The source data underlying Figs. 1c–g, 3, 4g–j, 4l–n, and Supplementary Fig. 1b–d are provided in the Source Data file.

## Source data

Source Data

## Code availability

Frequency analysis software is available on the Open Science Framework repository 10.17605/OSF.IO/5HBVN under an Apache License and can be downloaded at https://osf.io/5hbvn/. Matlab code used to examine and display the outputs from the Frequency Analysis program is also available at 10.17605/OSF.IO/5HBVN. Image registration software is freely available for non-commercial use on request. Instrument control and acquisition software has been customized to interface with hardware at the University of Rochester but is available to interested parties on request.

## References

[1] Lu Q, Ganjawala TH, Hattar S, Abrams GW, Pan Z-H (2018). A robust optomotor assay for assessing the efficacy of optogenetic tools for vision restoration. Invest. Ophthalmol. Vis. Sci..

[2] van Wyk M, Pielecka-Fortuna J, Löwel S, Kleinlogel S (2015). Restoring the ON switch in blind retinas: Opto-mGluR6, a next-generation, cell-tailored optogenetic tool. PLOS Biol..

[3] Berry MH (2017). Restoration of patterned vision with an engineered photoactivatable G protein-coupled receptor. Nat. Commun..

[4] Sengupta A (2016). Red‐shifted channelrhodopsin stimulation restores light responses in blind mice, macaque retina, and human retina. EMBO Mol. Med..

[5] Chaffiol A (2017). A new promoter allows optogenetic vision restoration with enhanced sensitivity in macaque retina. Mol. Ther..

[6] Klapoetke NC (2014). Independent optical excitation of distinct neural populations. Nat. Methods.

[7] Chen T-W (2013). Ultra-sensitive fluorescent proteins for imaging neuronal activity. Nature.

[8] Yin L (2011). Intravitreal injection of AAV2 transduces Macaque inner retina. Invest. Ophthalmol. Vis. Sci..

[9] Dalkara D (2009). Inner limiting membrane barriers to AAV-mediated retinal transduction from the vitreous. Mol. Ther..

[10] McGregor JE (2018). Functional architecture of the foveola revealed in the living primate. PLOS ONE.

[11] Russell S (2017). Efficacy and safety of voretigene neparvovec (AAV2-hRPE65v2) in patients with RPE65-mediated inherited retinal dystrophy: a randomised, controlled, open-label, phase 3 trial. Lancet.

[12] Dalkara D (2013). In vivo–directed evolution of a new adeno-associated virus for therapeutic outer retinal gene delivery from the vitreous. Sci. Transl. Med..

[13] Gaub BM, Berry MH, Holt AE, Isacoff EY, Flannery JG (2015). Optogenetic vision restoration using rhodopsin for enhanced sensitivity. Mol. Ther..

[14] Stockman A, Langendörfer M, Smithson HE, Sharpe LT (2006). Human cone light adaptation: from behavioral measurements to molecular mechanisms. J. Vis..

[15] Beyeler M, Rokem A, Boynton GM, Fine I (2017). Learning to see again: biological constraints on cortical plasticity and the implications for sight restoration technologies. J. Neural Eng..

[16] Bowen RW, Pokorny J, Smith VC (1989). Sawtooth contrast sensitivity: decrements have the edge. Vis. Res..

[17] Stasheff SF (2008). Emergence of sustained spontaneous hyperactivity and temporary preservation of off responses in ganglion cells of the retinal degeneration (rd1) mouse. J. Neurophysiol..

[18] Sekirnjak C (2011). Changes in physiological properties of rat ganglion cells during retinal degeneration. J. Neurophysiol..

[19] Marc RE, Jones BW, Watt CB, Strettoi E (2003). Neural remodeling in retinal degeneration. Prog. Retin. Eye Res..

[20] Barrett JM, Hilgen G, Sernagor E (2016). Dampening spontaneous activity improves the light sensitivity and spatial acuity of optogenetic retinal prosthetic responses. Sci. Rep..

[21] Sharma R (2016). In vivo two-photon fluorescence kinetics of primate rods and conestwo-photon fluorescence kinetics of primate rods and cones. Invest. Ophthalmol. Vis. Sci..

[22] Gray DC (2008). In vivo imaging of the fine structure of rhodamine-labeled Macaque retinal ganglion cells. Invest. Ophthalmol. Vis. Sci..

[23] Walters S (2018). Cellular-scale evaluation of induced photoreceptor degeneration in the living primate eye. Biomed. Opt. Express.

[24] Polyak, S.L. The retina: the anatomy and the histology of the retina in man, ape, and monkey, including the consideration of visual functions, the history of physiological optics, and the histological laboratory technique. University of Chicago Press, Chicago (1941).

