# Supplementary material for: Optogenetic restoration of retinal ganglion cell activity in the living primate
Source: Nat Commun. 2020 Apr 3;11:1703. doi: 10.1038/s41467-020-15317-6 (PMC7125151; doi:10.1038/s41467-020-15317-6)
Supplement: Supplementary file 1 — Supplementary Information [file 41467_2020_15317_MOESM1_ESM.pdf]

Supplementary information file

Optogenetic restoration of retinal ganglion cell activity in the living primate

Juliette E. McGregor, Tyler Godat, Kamal R. Dhakal, Keith Parkins, Jennifer M. Strazzeri, Brittany A. Bateman, William S. Fischer, David R. Williams, William H. Merigan.

Containing:

Supplementary Figure 1

Supplementary Figure 2

Supplementary Figure 3

Supplementary Table 1

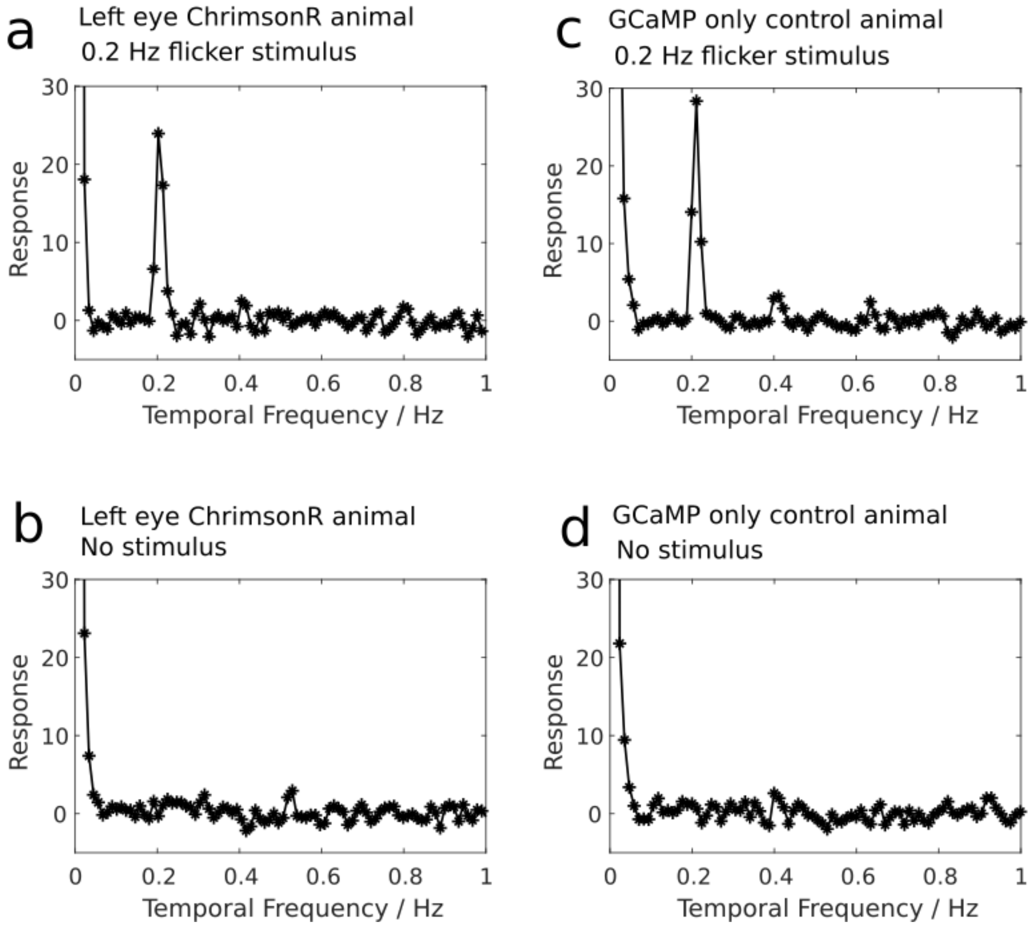

Supplementary Figure 1: The RGC response of a ChrimsonR + GCaMP6s eye, and a GCaMP6s only control eye to a 0.2Hz pan retinal flicker stimulus is similar, confirming that the control eye is normally responsive to photoreceptor stimulation and provides an appropriate control. a). The response of the same cells in Figure 1c of the ChrimsonR + GCaMP6s eye to a pan retinal 0.2Hz flicker stimulus 5 months after injection. **b.)** No response above noise is observed at 0.2 Hz from the same cells when no periodic stimulus is provided to the eye containing ChrimsonR **c).** The response of the same cells in Figure 1d of the GCaMP6s only control eye, to a pan retinal 0.2Hz flicker stimulus 5 months after injection. **d).** No response above noise is observed at 0.2 Hz from the same cells when no periodic stimulus is provided to the eye containing GCaMP6s only. Representative data shown, similar results were obtained in 5 imaging sessions. Source data are provided as a Source Data file.

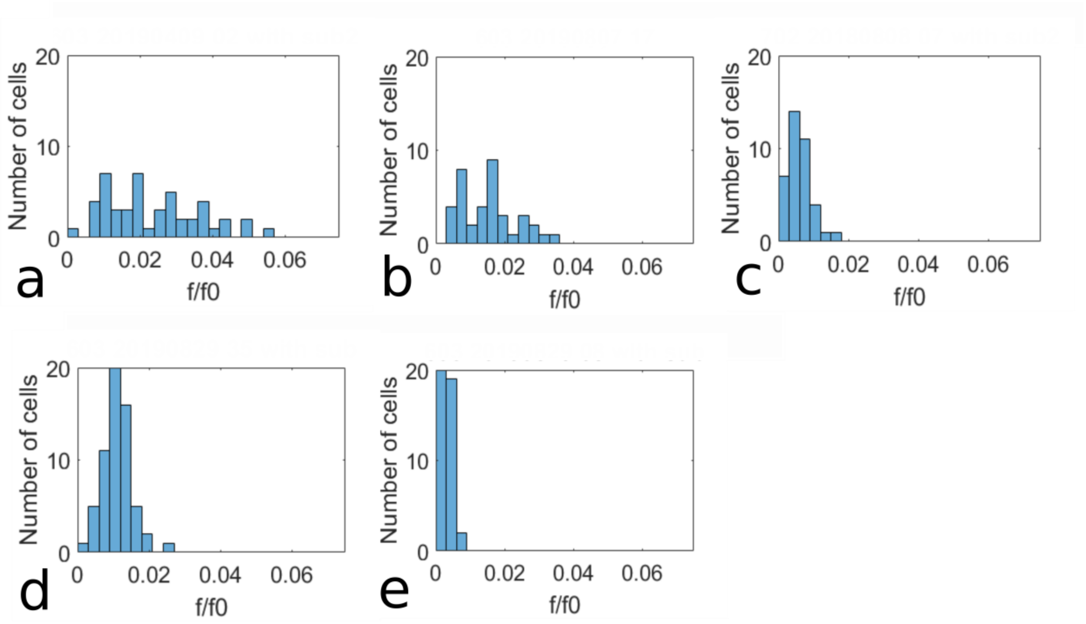

Supplementary Figure 2: F/F0 histograms showing individual cellular responses generating the mean data shown in panels 1 c-g corresponding to histograms a-e respectively. a and b show a higher range of responsivities relative to the g camp only control eye c. These data were taken at time points over 5 months after intravitreal injection in animals that had received 1 week of immune suppression prior to the first intravitreal injection. Panels d and e correspond to a data trial in the left eye of animal 4 and a control trial in the right eye of animal 4. There is a similar difference in the distributions of the f/f0 responses between the trial and control eyes with all values being lower than panels a-c, this is likely a reflection of the increased brightness in this animal and not necessarily a reflection of decreased responsivity. Increased brightness may have been caused by the earlier time point, (7 and 9 weeks for d and e respectively) or the longer period of immune suppression (2 weeks before the first injection). The relationship between f and f0 is not necessarily linear or straightforward in the retina *in vivo* and care should be taken when interpreting this metric.

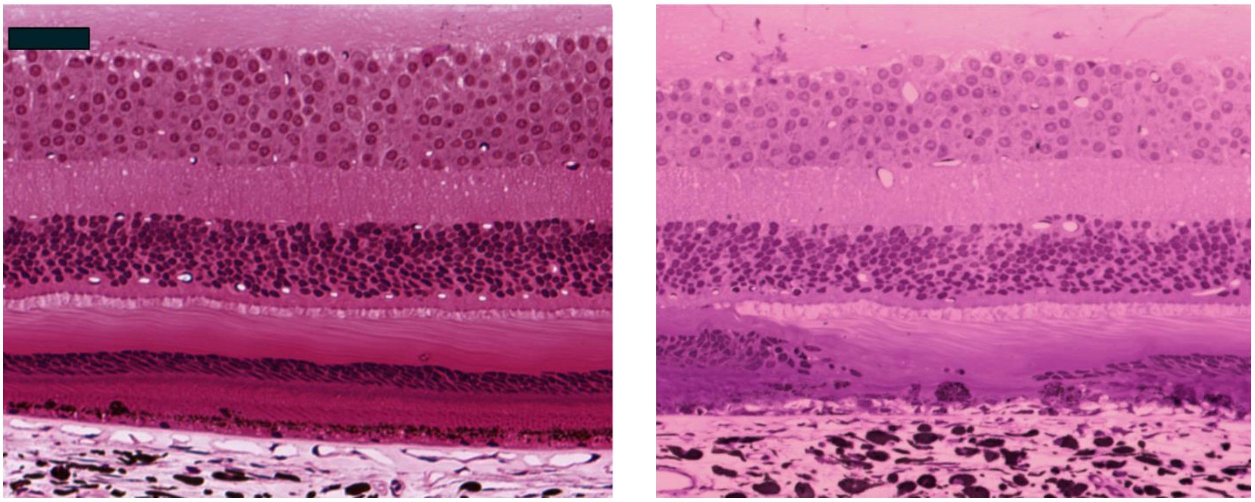

Supplementary Figure 3: Histological evidence of photoreceptor loss. Scale bar 50µm. Left panel: Normal retinal layers in a neighbouring unexposed region of the retina. The outer nuclear layer is continuous and intact. Right panel: Layers of the retina following ultrafast laser exposure delivered through the adaptive optics system. The loss of the outer nuclear layer in this area is consistent with the loss of functional photoreceptors in this area. Histology taken from a single lesion in one animal at this exposure setting.

	Neutralizing antibodies to AAV2 (in blood serum)	GCaMP6s titre (vg/ml)	GCaMP6s volume (µl)	ChrimsonR titre (vg/ml)	ChrimsonR volume/ µl
Animal 1 (Histology: Treated)<br>OS, Male	1:8	1.94×10^{13}	75	1.05×10^{12}	75
Animal 2 (Recording: Treated) OD, Male	1:2	1.94×10^{13}	75	1.05×10^{12}	75
Animal 2<br>(Recording: Treated) OS, Male	1:2	1.94×10^{13}	50	1.05×10^{12}	50

Animal 3<br><br>(Recording: Control)<br><br>OS, Female	1:25	$1.94x10^{13}$	100	-------	------
Animal 4<br><br>(Recording: Control)<br>OD, Female	< 1:5	$1.90x10^{13}$	75	$1.05x10^{12}$	------
Animal 4<br><br>(Recording: Treated)<br>OS, Female	< 1:5	$1.9x10^{13}$	75	$1.05x10^{12}$	75
Animal 5 (Histology)<br><br>OS, Male	Femtosecond laser exposure only				

Supplementary Table 1: List of all eyes involved in the study including the antibody status of the animal prior to injection and the viral doses injected into each eye.
