# Supplementary material for: Optogenetic restoration of retinal ganglion cell activity in the living primate
Source: Nat Commun. 2020 Apr 3;11:1703. doi: 10.1038/s41467-020-15317-6 (PMC7125151; doi:10.1038/s41467-020-15317-6)
Supplement: Supplementary file 2 — Reporting Summary [file 41467_2020_15317_MOESM2_ESM.pdf]

natureresearch

Corresponding author(s): Juliette McGregor and William Merigan

Last updated by author(s): 2/4/2020

Reporting Summary

Nature Research wishes to improve the reproducibility of the work that we publish. This form provides structure for consistency and transparency in reporting. For further information on Nature Research policies, see Authors & Referees and the Editorial Policy Checklist .

Statistics

For all statistical analyses, confirm that the following items are present in the figure legend, table legend, main text, or Methods section.

n/a	Confirmed	
☐	☒	The exact sample size (*n*) for each experimental group/condition, given as a discrete number and unit of measurement
☐	☒	A statement on whether measurements were taken from distinct samples or whether the same sample was measured repeatedly
☒	☐	The statistical test(s) used AND whether they are one- or two-sided<br>*Only common tests should be described solely by name; describe more complex techniques in the Methods section.*
☒	☐	A description of all covariates tested
☐	☒	A description of any assumptions or corrections, such as tests of normality and adjustment for multiple comparisons
☐	☒	A full description of the statistical parameters including central tendency (e.g. means) or other basic estimates (e.g. regression coefficient) AND variation (e.g. standard deviation) or associated estimates of uncertainty (e.g. confidence intervals)
☒	☐	For null hypothesis testing, the test statistic (e.g. *F*, *t*, *r*) with confidence intervals, effect sizes, degrees of freedom and *P* value noted<br>*Give P values as exact values whenever suitable.*
☒	☐	For Bayesian analysis, information on the choice of priors and Markov chain Monte Carlo settings
☒	☐	For hierarchical and complex designs, identification of the appropriate level for tests and full reporting of outcomes
☒	☐	Estimates of effect sizes (e.g. Cohen's *d*, Pearson's *r*), indicating how they were calculated

Our web collection on statistics for biologists contains articles on many of the points above.

Software and code

Policy information about availability of computer code

Data collection	Custom instrument control and image acquisition code was developed to interface with specialized AOSLO hardware at the University of Rochester. The code is available on request.
Data analysis	Image registration was performed using custom software based on cross correlation, this software is available for non-commercial uses on request. Custom code was developed to perform Fourier analysis on the fluorescence videos. Frequency analysis software is available on the Open Science Framework repository DOI 10.17605/OSF.IO/5HBVN under an Apache License and can be downloaded at https://osf.io/5hbvn/. Matlab code used to examine and display the outputs from the Frequency Analysis programs can be downloaded from the same location.

For manuscripts utilizing custom algorithms or software that are central to the research but not yet described in published literature, software must be made available to editors/reviewers. We strongly encourage code deposition in a community repository (e.g. GitHub). See the Nature Research guidelines for submitting code & software for further information.

Data

Policy information about availability of data

All manuscripts must include a data availability statement. This statement should provide the following information, where applicable:

- Accession codes, unique identifiers, or web links for publicly available datasets
- A list of figures that have associated raw data
- A description of any restrictions on data availability

All raw data is available on request. The source data underlying Figs 1c-g, 3, 4g-j, 4l-n, and Supplementary Figs S1b-d are provided in the Source Data file.

Field-specific reporting

Please select the one below that is the best fit for your research. If you are not sure, read the appropriate sections before making your selection.

- [x] Life sciences
- [ ] Behavioural & social sciences
- [ ] Ecological, evolutionary & environmental sciences

For a reference copy of the document with all sections, see nature.com/documents/nr-reporting-summary-flat.pdf

Life sciences study design

All studies must disclose on these points even when the disclosure is negative.

Sample size	No sample size calculations were performed. The statistical detectability (sensitivity index) of the signal in each treated eye was large enough that the study could be completed using in vivo recordings from three treated eyes and two control eyes without the need for statistical tests to determine significance between groups. Multiple experiments producing multiple lines of evidence in support of the hypothesis were also conducted in each eye. In primate research expense and ethical considerations mean that studies should be designed and performed with the lowest number of animals possible. An additional two eyes were used for histology.
Data exclusions	No data was excluded
Replication	In vivo imaging data was collected from three treated eyes plus two control eyes. Trials have been repeated in the same retinal locations over months with both the same and differing stimulus powers and at different wavelengths. Within each imaging session data was collected from different imaging locations around the fovea and up to 4 repeated trials were collected at the same location depending on light exposure constraints. All attempts at replication were successful.
Randomization	Statistical randomization was not relevant to this study due to the low number of individuals. Two eyes from one male animal and one eye from a female animal received the therapeutic. To allow direct comparison between the control and therapeutic condition in the same individual, the second eye of the same female was used as a control. Recordings were made from the control eye of an additional female animal to confirm the result. Allocation into the therapeutic and control groups was constrained to some extent by the availability of animals at the time.
Blinding	Researchers were not blinded to the identities of the control and test eyes. This was because the same person designed and set up the experiment, collected the data and analyzed it. As such, to perform the experiment they needed to be aware of the identity of the animal, distinctive retinal features for navigation and the trials conducted, making blinding difficult in this case.

Reporting for specific materials, systems and methods

We require information from authors about some types of materials, experimental systems and methods used in many studies. Here, indicate whether each material, system or method listed is relevant to your study. If you are not sure if a list item applies to your research, read the appropriate section before selecting a response.

Materials & experimental systems

n/a	Involved in the study	
[x]	[ ]	Antibodies
[x]	[ ]	Eukaryotic cell lines
[x]	[ ]	Palaeontology
[ ]	[x]	Animals and other organisms
[x]	[ ]	Human research participants
[x]	[ ]	Clinical data

Methods

n/a	Involved in the study	
[x]	[ ]	ChIP-seq
[x]	[ ]	Flow cytometry
[x]	[ ]	MRI-based neuroimaging

Animals and other organisms

Policy information about studies involving animals; ARRIVE guidelines recommended for reporting animal research

Laboratory animals	This study involved 5 Macaca fascicularis, three male, two female, aged 6, 4, 7, 6 and 4 years respectively at the start of the study. Two animals have been euthanised, three are involved in ongoing vision restoration projects.
Wild animals	The study did not involve wild animals
Field-collected samples	The study did not involve field collected samples
Ethics oversight	The protocol was approved by the University Committee on Animal Resources of the University of Rochester (PHS assurance number D16-00188(A3292-01))

Note that full information on the approval of the study protocol must also be provided in the manuscript.
